# Supplementary material for: Human RIF1 and protein phosphatase 1 stimulate DNA replication origin licensing but suppress origin activation
Source: EMBO Rep. 2017 Jan 11;18(3):403–19. doi: 10.15252/embr.201641983 (PMC5331243; doi:10.15252/embr.201641983)
Supplement: Supplementary file 1 — Appendix [file EMBR-18-403-s001.pdf]

Appendix figures for Hiraga et al., "**Human RIF1 and Protein Phosphatase 1 stimulate DNA replication origin licensing but suppress origin activation**"

Table of contents

Page 2: Appendix Figure S1**.** PP1 depletion by siRNA

Page 3: Appendix Figure S2**.** Effect of depleting PP1 on MCM4 phosphorylation

Page 4: Appendix Figure S3**.** Effect of RIF1 on cellular sensitivity against DDK inhibition

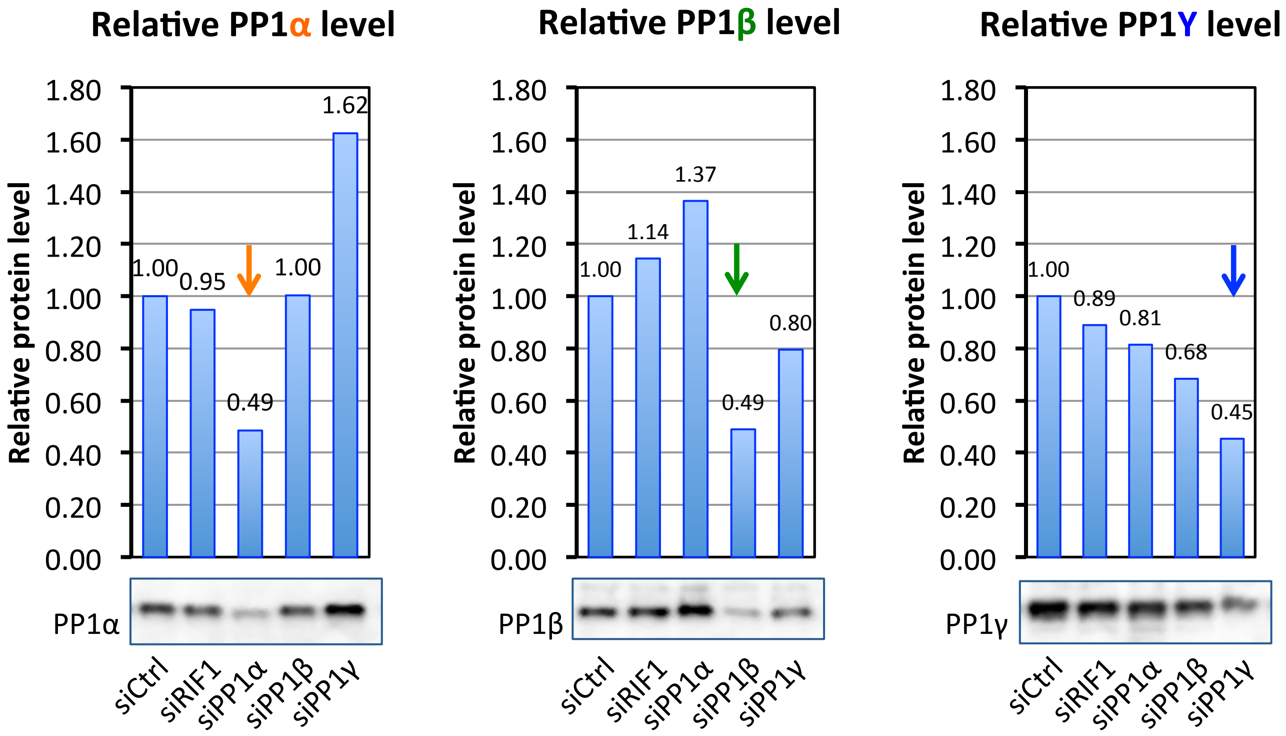

Appendix Fig S1. PP1 depletion by siRNA

Quantification of band intensities relative to the control siRNA (siCtrl) samples in the western blots presented in Fig 2B (i). The relevant western panels are reproduced below the plots for clarity. Protein load was normalized by total protein. Note that depletion of each individual isoform caused an apparent compensatory increase in others (for example, PP1α increases when PP1γ is depleted; see PP1γ siRNA lane in the PP1α panel).

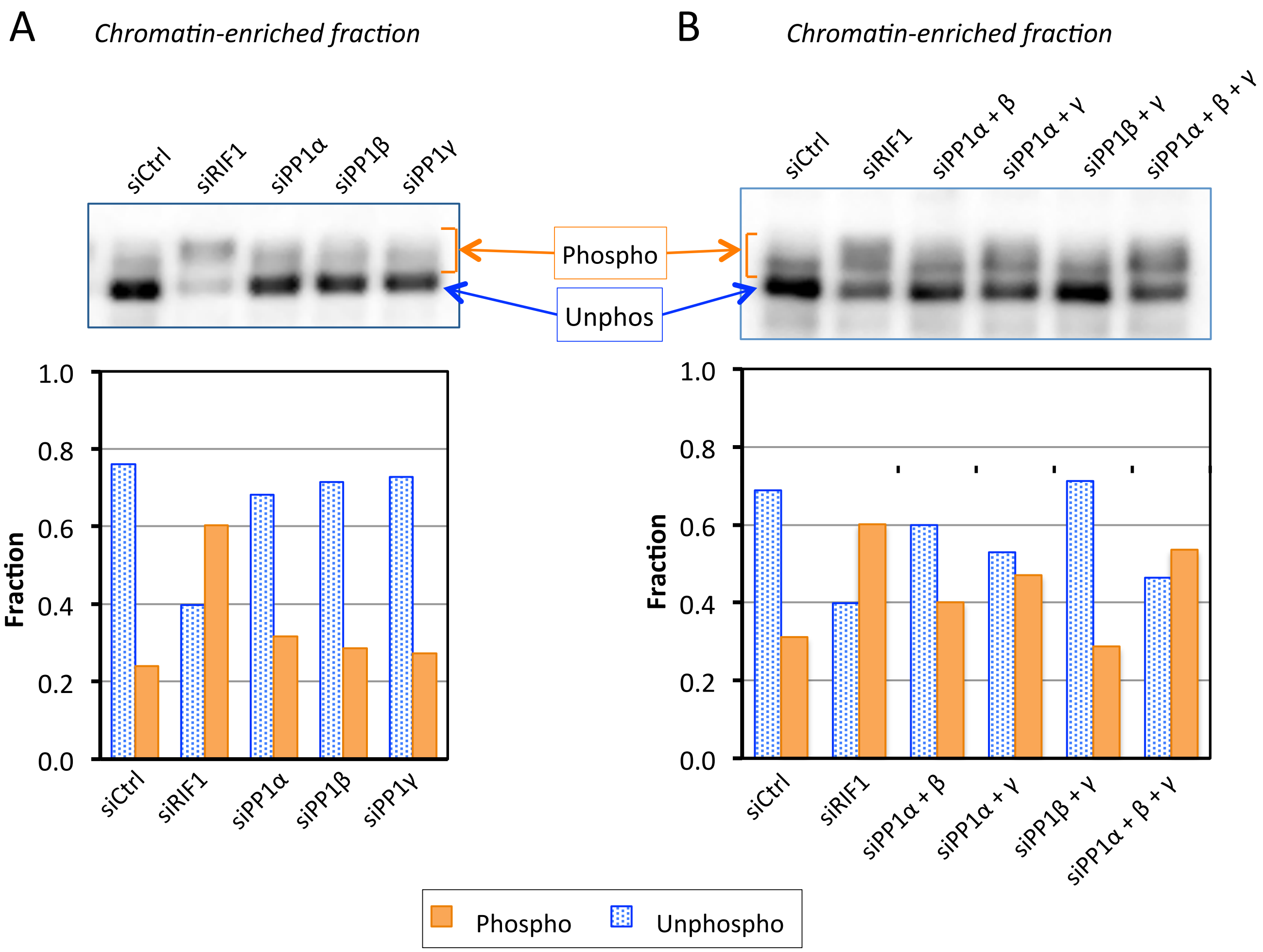

Appendix Fig S2. Effect of depleting PP1 on MCM4 phosphorylation.

Quantification of the effect on MCM4 phosphorylation status of depleting single PP1 isoforms (A) and combinations of PP1 isoforms (B). Relative intensities of unphosphorylated and phosphorylated MCM4 bands in the western blots presented in Fig 2B are plotted. The relevant western panels are reproduced above the plots for clarity. Protein load was normalized by total protein.

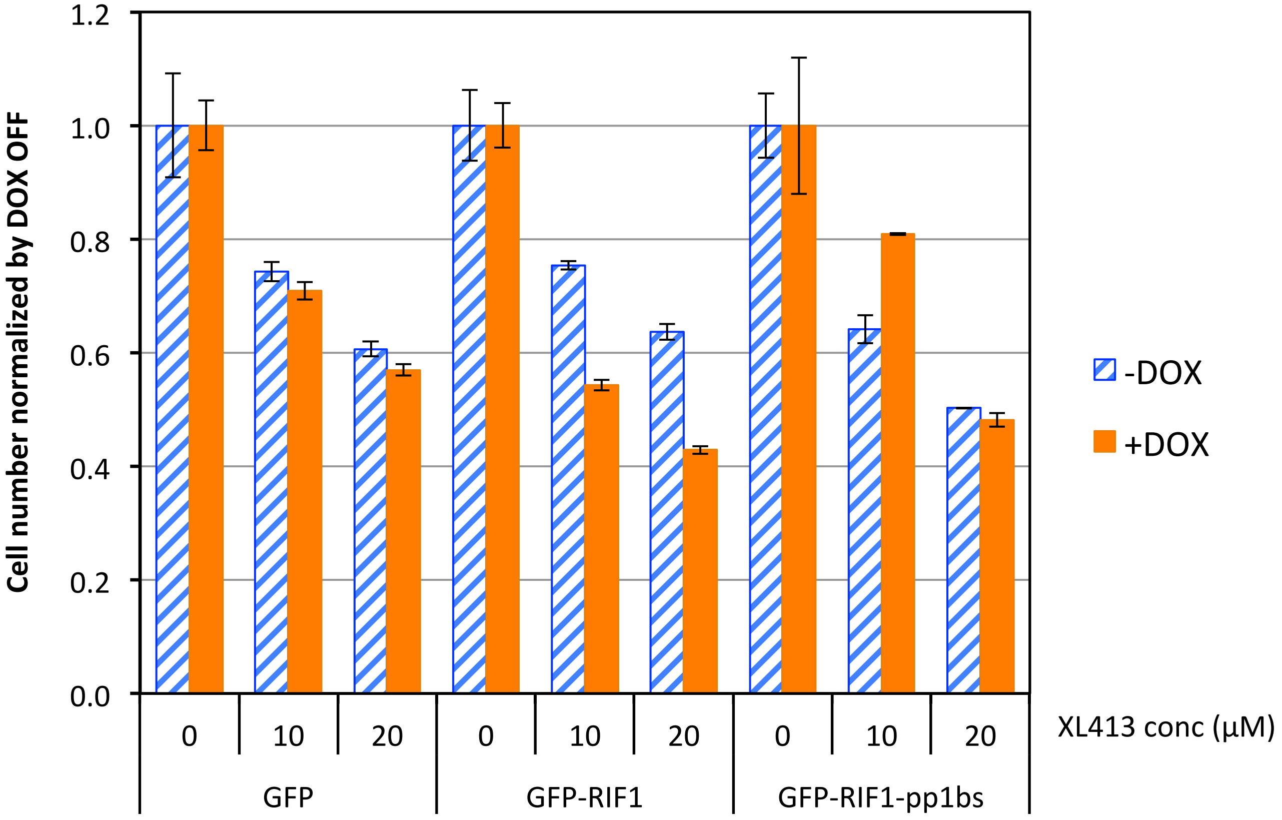

Appendix Fig S3. Effect of RIF1 on cellular sensitivity against DDK inhibition.

PP1 interaction is required for RIF1 to sensitize cells to DDK inhibition. Cell lines containing GFP, GFP-RIF1, or GFP-RIF1-pp1bs constructs were seeded in white 96-well plates at 4,000 cells/well, and cultured for 3 days with or without DOX and in the presence of XL413 at the indicated concentration. Relative numbers of metabolically active cells per well were assessed using CellTiter-Glo. Error bars indicate the SEM of 3 biological replicates.
