# Supplementary material for: Human RIF1 and protein phosphatase 1 stimulate DNA replication origin licensing but suppress origin activation
Source: EMBO Rep. 2017 Jan 11;18(3):403–19. doi: 10.15252/embr.201641983 (PMC5331243; doi:10.15252/embr.201641983)
Supplement: Supplementary file 2 — Expanded View Figures PDF [file EMBR-18-403-s002.pdf]

Expanded View Figures

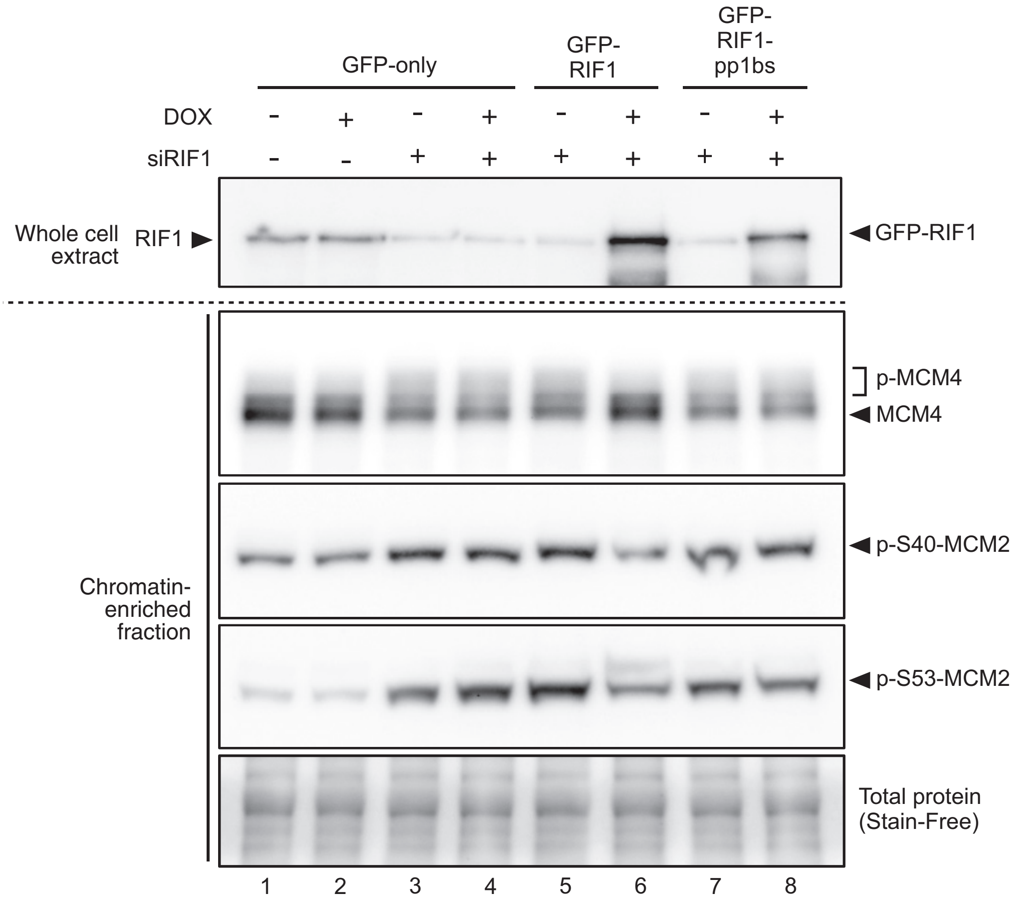

Figure EV1. Effect of RIF1 and its PP1 interaction on phosphorylation status of MCM proteins.
Phosphorylation status of MCM4 and MCM2 was analyzed as in Fig 2B, in a biological duplicate experiment.

 © 2017 The Authors

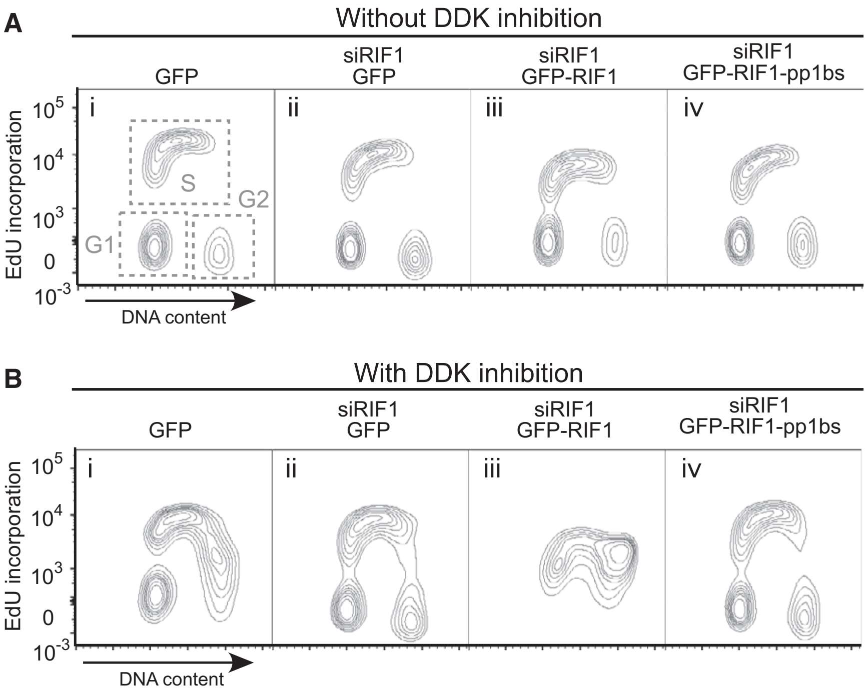

Figure EV2. Effect of RIF1 on cellular DNA synthesis.

A Effect of depletion followed by ectopic expression of RIF1 on DNA synthesis and cell cycle distribution, showing two-dimensional plots of samples in Fig 3B and C. Flp-In T-REx 293 cells with stable GFP, GFP-RIF1, or GFP-RIF1-pp1bs constructs were depleted for endogenous RIF1 by siRNA, and 1 day later, GFP, GFP-RIF1, or GFP-RIF1-pp1bs was induced. Further 2 days later, cells were pulse-labeled with 20 μM EdU for 15 min prior to preparation of cells for flow cytometry analysis of DNA content and EdU incorporation. Note the *y*-axis scale is bi-exponential.

B Effect of depletion and ectopic expression of RIF1 on DNA synthesis limited by XL413 addition. Cells were prepared as in (A) but in the presence of 10 μM XL413, added at the same time as DOX induction.

Figure EV3. RIF1 promotes origin licensing via protecting ORC1 from degradation.

A Rif1 removal or loss of Rif1-PP1 interaction compromises chromatin association of MCM4 protein. The abundance of chromatin-associated MCM4 (phosphorylated + unphosphorylated) was measured in Western blots and plotted relative to the control condition (i.e., Flp-In T-REx 293 cell line containing GFP construct without DOX induction). Bars plot the average value obtained in two biologically independent experiments, with the values obtained from each experiment indicated by filled circles.

B Specimen image of permeabilized HeLa-derived cells expressing GFP-ORC1 and mCherry-PCNA, showing that either GFP-ORC1 or mCherry-PCNA is chromatin-associated but not both. GFP-ORC1 mCherry-PCNA HeLa cells were permeabilized with 0.1% NP-40, fixed for 10 min by 1% formaldehyde, and stained with 0.5 μg/ml DAPI. DAPI-stained DNA (blue), GFP-ORC1 (green), and mCherry-PCNA (red) are shown in pseudo-color. Scale bar indicates 30 μm.

C RIF1 is required for protection of GFP-ORC1. Flow cytometry quantification of cell cycle-specific association of GFP-ORC1 and mCherry-PCNA in GFP-ORC1 mCherry-PCNA HeLa cells. The cells were transfected with either control siRNA (siCtrl) or siRNA against RIF1 (siRIF1), followed by 3 days of incubation before flow cytometry analysis. Note the *y*-axis scale of mCherry-PCNA plot is bi-exponential.

D Proportion of G1-phase cells showing “high-ORC1” and “high-MCM3” chromatin association. The fractions of G1-phase cells with “high-FLAG-ORC1”, “high-GFP-ORC1”, and “high-MCM3” chromatin association were measured as shown in Fig 4C and D, and panel (C). Results show the means of two, five, and three experiments for FLAG-ORC1, GFP-ORC1, and MCM3, respectively. Filled circles indicate the values obtained from two independent experiments for FLAG-ORC1, with error bars indicating the SEM in other experiments.

E RIF1 is required for full loading of MCM3 on chromatin in the FLAG-ORC1 293 cell line. Chromatin-associated MCM3 protein was analyzed by flow cytometry in the same set of cells as in Fig 4D and E.

F Transient treatment with tautomycetin does not affect cell cycle distribution. Histograms indicate the DNA content of cells analyzed in Fig 5A and B.

G In RIF1-depleted cells proteasome inhibition rescues the ORC1 protection defect and restores ORC1 chromatin association. Control and RIF1-depleted GFP-ORC1 mCherry-PCNA HeLa cells were incubated for 4 h in the presence of 5 μM MG-132 and analyzed for abundance of chromatin-associated GFP-ORC1 protein as in panel (C).

© 2017 The Authors

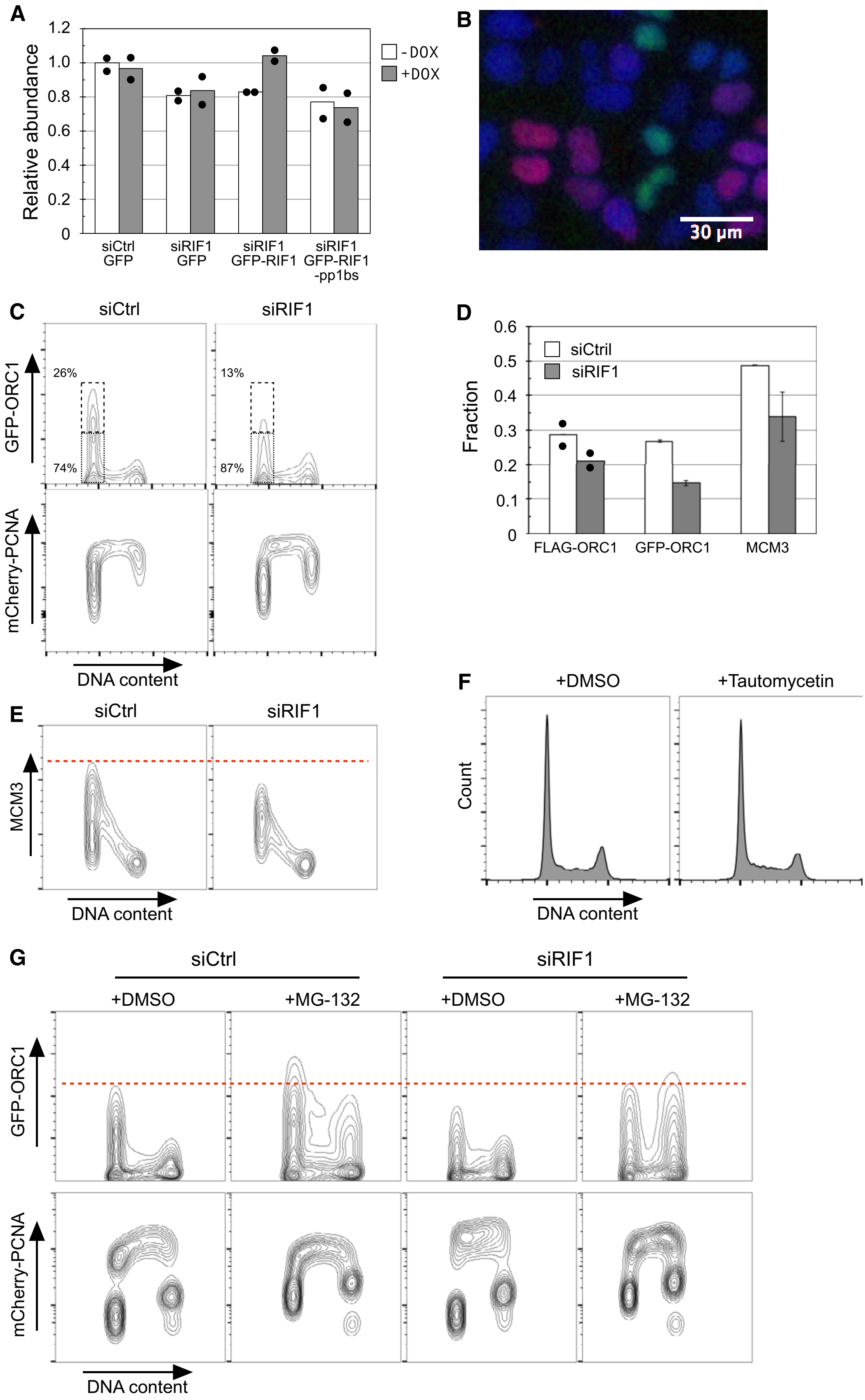

Figure EV3.

 © 2017 The Authors

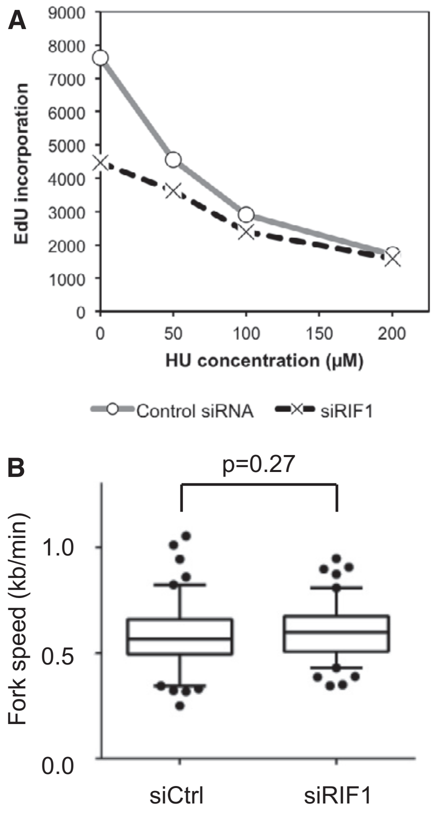

Figure EV4. Interorigin distance is affected by RIF1.

A Defective DNA synthesis in RIF1-depleted cells in the presence of low HU concentrations. siCtrl- and siRIF1-treated HEK293 cells were incubated for 4.5 h with the indicated concentration of HU and in the final 30 min EdU-labeled to assess the cellular DNA synthesis rate, measured by flow cytometry analysis.

B Loss of RIF1 does not affect replication fork rate. Flp-In T-REx cells transfected with siCtrl (left) or siRIF1 (right) were pulse-labeled sequentially with CldU and IdU, and replication fork rate was determined based on the IdU and CldU track lengths. The results are presented as box-and-whisker plots. Whiskers indicate percentiles 5–95, and dots indicate outliers. *P*-value was calculated by *t*-test. n = 100 for each condition.

© 2017 The Authors

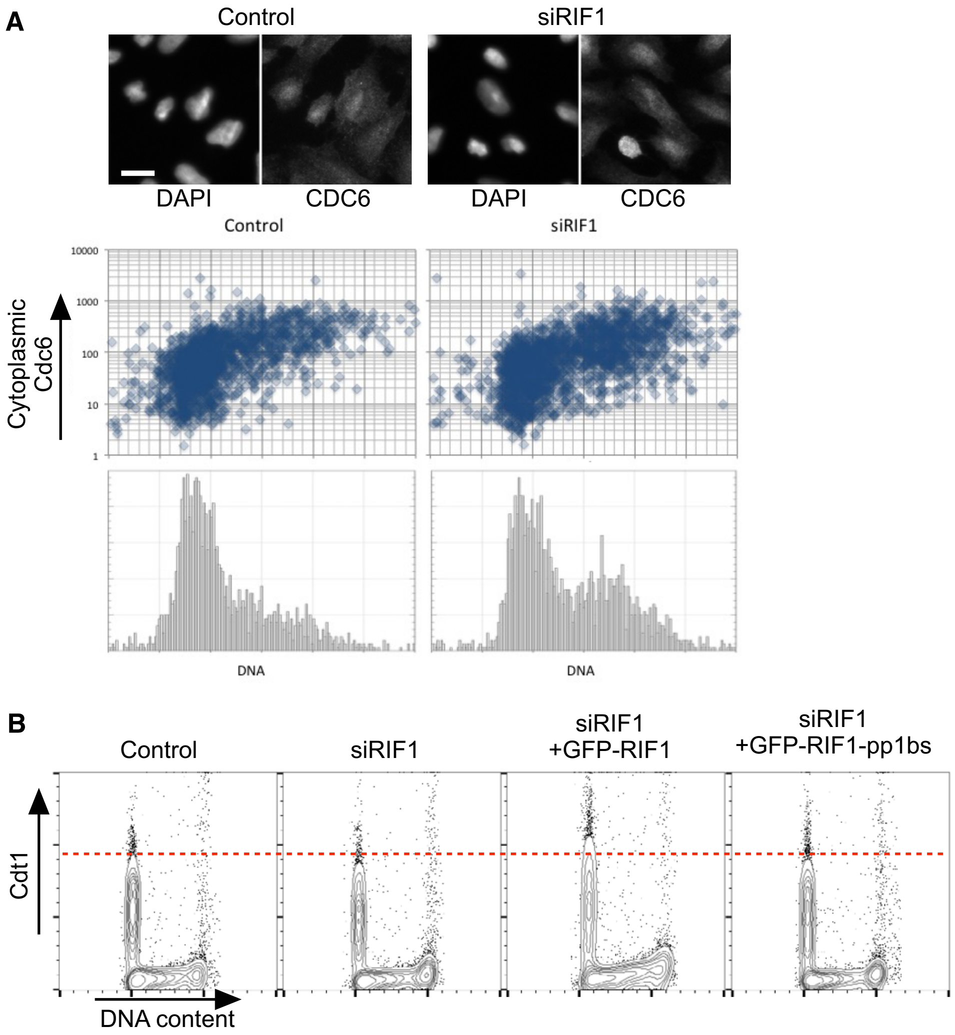

Figure EV5. RIF1 does not control Cdt1 and Cdc6.

A RIF1 does not control the cell cycle-specific localization of Cdc6 protein. Subcellular localization of Cdc6 protein was previously shown to be cell cycle regulated through its phosphorylation by CDK, which leads to export of Cdc6 protein from the nucleus to the cytoplasm [47]. To test whether Rif1 removal affects Cdc6 regulation, subcellular localization of Cdc6 was analyzed by immunofluorescence microscopy in U2OS cells treated with either siCtrl or siRIF1. Abundance of cytoplasmic Cdc6 and DNA content was measured for more than 1,700 cells under each condition using CellProfiler software as follows. Adaptive thresholding was applied to the blue (DAPI-stained nuclear DNA) and green (anti-Cdc6 stain) channels to identify areas corresponding to individual nuclei and cells, respectively. The cytoplasmic area was then defined by excluding the nuclear area from the cell area. For each cell, the integrated intensity in the green (Cdc6) channel was then measured separately for the cytoplasmic area, the nuclear area, and the whole cell. Integrated intensity in the blue channel was measured for each nuclear area to obtain a cellular DNA content value. Cells at the edge of images were excluded. Scatter plots show cytoplasmic Cdc6 abundance (*y*-axis; $\log_{10}$ scale) against DNA content (*x*-axis), while the histograms (lower panels) show DNA content of the corresponding cells. Representative images are presented at the top. The scale bar indicates 30 μm.

B RIF1 has only a minor effect on Cdt1 stability. Degradation of Cdt1 protein is regulated in a similar way to ORC1: outside G1 phase, Cdt1 is phosphorylated and degraded [48]. The effect of depletion followed by ectopic expression of RIF1 on Cdt1 abundance was tested by flow cytometry. Flp-In T-REx 293 cells with stable GFP, GFP-RIF1, or GFP-RIF1-pp1bs constructs were depleted for endogenous RIF1 by siRNA, and 1 day later GFP, GFP-RIF1, or GFP-RIF1-pp1bs was induced. After further 3 days, cells were prepared for flow cytometry and abundance of Cdt1 protein per cell was analyzed by flow cytometry.

© 2017 The Authors
